# Attrition in an Online Loneliness Intervention for Adults Aged 50 Years and Older: Survival Analysis

**DOI:** 10.2196/13638

**Published:** 2019-07-24

**Authors:** Tamara Bouwman, Theo van Tilburg, Marja Aartsen

**Affiliations:** 1 Department of Sociology Faculty of Social Sciences Vrije Universiteit Amsterdam Amsterdam Netherlands; 2 Norwegian Social Research Oslo Metropolitan University Oslo Norway

**Keywords:** online intervention, loneliness, attrition, coping, engagement, older adults

## Abstract

**Background:**

Online interventions can be as effective as in-person interventions. However, attrition in online intervention is high and potentially biases the results. More importantly, high attrition rates might reduce the effectiveness of online interventions. Therefore, it is important to discover the extent to which factors affect adherence to online interventions. The setting for this study is the online Friendship Enrichment Program, a loneliness intervention for adults aged 50 years and older.

**Objective:**

This study examined the contribution of severity of loneliness, coping preference, activating content, and engagement in attrition within an online intervention.

**Methods:**

Data were collected from 352 participants in an online loneliness intervention for Dutch people aged 50 years and older. Attrition was defined as not completing all 10 intervention lessons. The number of completed lessons was assessed through the management system of the intervention. We tested 4 hypotheses on attrition by applying survival analysis (Cox regression).

**Results:**

Of the 352 participants who subscribed to the intervention, 46 never started the introduction. The remaining 306 participants were divided into 2 categories: 73 participants who did not start the lessons of the intervention and 233 who started the lessons of the intervention. Results of the survival analysis (n=233) showed that active coping preference (hazard ratio [HR]=0.73), activating content (HR=0.71), and 2 indicators of engagement (HR=0.94 and HR=0.79) lowered attrition. Severity of loneliness was not related to attrition.

**Conclusions:**

To reduce attrition, developers of online (loneliness) interventions may focus on stimulating active behavior within the intervention.

## Introduction

During recent years, the number of online interventions has increased rapidly. Online interventions offer possibilities for reaching more participants [1], are more cost-effective, and are less prone to stigma than in-person interventions [2]. Another advantage is that participation in online interventions can be undertaken as per participants’ preferred pace, as opposed to the fixed structure of in-person group interventions. Furthermore, online interventions can be as beneficial as in-person interventions [3,4]. They are often self-guided, that is, there is no contact between the participant and a coach or therapist [5]. Eysenbach [6] points out that online interventions are characterized by high attrition, which is also stressed in later studies [7]. During intervention trials, participants drop out quite easily, without further consequences. The understanding of factors associated with attrition is limited. In this study, we examined factors that may be associated with attrition in an online self-guided loneliness intervention for older adults. Loneliness is nowadays considered as 1 of the main social problems in society [8]. Fighting loneliness contributes to the improvement of individual well-being and lowers the risk of poor health and early mortality.

It is important to gain more insight into online intervention attrition for 2 reasons. First, dropout attrition, which refers to participants dropping out of the study but not leaving the intervention, affects the study’s effectiveness [6]. If dropout attrition is selective, study results are biased. Moreover, dropout attrition affects the statistical power of the study. Second, participants who discontinue the intervention during the course of the intervention (nonusage attrition [6]) do not benefit from the intervention optimally. Although Eysenbach’s paper [6] was published several years ago, his ideas regarding attrition are still topical today [7,9]. One way to improve the effectiveness of the intervention is to reduce nonusage attrition. This study aimed to provide more insight into factors affecting nonusage attrition in online self-guided interventions.

We examined several factors that might be related to nonusage attrition. Although research on attrition in online interventions is still limited, we have expectations with respect to nonusage attrition. First, participants who are severely lonely may be most likely to complete the intervention. The review of studies on the severity of the target problem as a factor in intervention dropout by Melville et al [10] reveals that participants with less severe problems are more likely to drop out, as they may be less motivated to invest time and effort into working on the problem. We, therefore, hypothesized that lonelier participants are more likely to complete the intervention (hypothesis 1).

Coping preference may be another factor affecting attrition. We distinguish between active and regulative coping [11]. People who have a preference for active coping want to tackle the loneliness problem by changing the undesirable situation, for example, by engaging in social activities. This suggests that people who tend to use active coping more often keep trying and persevere in completing the intervention (hypothesis 2a). In contrast, people with a preference for regulative coping do not attempt to deal with the problem itself. Instead, they try to minimize the emotional consequences of the problem by, for example, distracting oneself from the undesired situation. We, therefore, expect that participants with a preference for regulative coping are more likely to drop out (hypothesis 2b).

The intervention content itself can stimulate more active responses to address the problem at hand [12]. Assignments involving activity directed at a desired goal, when completed successfully, may offer rewards (such as satisfaction) that encourage participants to stay in the intervention longer. Stimulating active coping through the intervention’s content increases the likelihood of completing the intervention (hypothesis 3).

In online interventions, there is often little or no supervision on usage, and it is not always clear to which extent participants use the intervention as intended [6]. In addition, the intervention used in this study has no supervision. In other types of interventions, for example, drug trials, participants are supervised closely because quitting can have (health) consequences related to the medication that is being tested. Attrition is likely a consequence of lack of user engagement [7,13,14]. An early sign of this lack of engagement is that a participant hesitates to follow through after signing up or is slow in fulfilling tasks in the intervention [6]. A sign of sufficient engagement would be the enthusiasm with which participants start the intervention, for example, in terms of compliance with the intervention. We, thus, expect that participants showing high engagement at the start of the intervention are more likely to complete the intervention than participants with low initial engagement (hypothesis 4).

Other user-related characteristics that are associated with attrition in online interventions have been identified. Melville et al [10] suggest that having a partner reduces the likelihood of attrition, which may indicate that support, for example, provided by the partner, reduces the dropout rate [15]. However, an association between having a partner and dropout was not found in a meta-analysis [5]. Self-efficacy may also be related to attrition in online interventions, but the effects are ambiguous. A study by Glasgow et al [14] demonstrates that participants with (topic-specific) high self-efficacy are less likely to be engaged with the intervention on an ongoing basis and have a higher likelihood of not participating in the follow-up observation. In contrast, Wangberg et al [16] show that higher self-efficacy is related to more intense usage of the intervention. These contradictory findings necessitate further study of the effect of self-efficacy on attrition. Proficiency with information and communication technologies (ICTs) may also be related to attrition. Mathew et al show that participants with good internet skills are more likely to use an online physical activity intervention [17]. Finally, some studies show lower dropout rate among females, participants in older age categories, and participants with a high educational level [5,16].

The setting for this study is the online Friendship Enrichment Program (oFEP), a loneliness intervention for adults aged 50 years and older [18]. To the best of our knowledge, no other studies specifically examined attrition in online loneliness interventions. Therefore, this study aimed to discover the extent to which the abovementioned factors affect adherence to an online loneliness intervention.

## Methods

### Design of the Intervention and Study

The oFEP is an intervention for people aged 50 years and older. It is a Web-based adaptation of a successful in-person intervention [19]. The oFEP is an 11-week intervention consisting of an introductory lesson followed by 2 blocks of 5 lessons. The intervention was designed with the intention that participants complete 1 lesson each week. Participants could delay the start of a lesson if that was more convenient for them (eg, because of a vacation or hospitalization). One of the assumptions behind the intervention was that, to fully benefit from the intervention, it is best to complete all the lessons. The website of the intervention is in Dutch and designed in such a way that the website and the lessons can function on various types of devices. A previous study on the oFEP [18] showed that the program alleviates the loneliness of its participants to some extent. The study by Bouwman et al gives a more detailed description of the program [18].

We collected data at 4 time points: before the start of the intervention (T_1_), after the first block of the intervention (T_2_), at the end of the intervention (T_3_), and 1 year after the intervention (T_4_). The questionnaires at T_1_ and T_4_ were identical, and the questionnaires at T_2_ and T_3_ were shortened versions of the same questionnaire. Besides loneliness, other concepts, such as social self-efficacy, self-esteem, and participation, were measured. Participation in the intervention automatically meant participation in the study, which was communicated to the participants before signing up for the intervention. Starting to answer a questionnaire was a requirement to gain access to the next lesson. However, item nonresponse did not have consequences for participation. For this study, we used the baseline questionnaire and activity logs of intervention usage obtained through the management system of the intervention.

We identified 3 phases during which participants could drop out of the intervention. The first was directly after signing up for the intervention and before providing any information. Participants who dropped out in this phase never started the intervention and did not fill out any questionnaire (n=46). The second phase was before one participates in lessons. These participants filled out the baseline questionnaire and completed the intervention’s introduction (n=73). The third phase was during the actual participation in the intervention (n=151). This category included all participants who completed between 1 and 10 lessons of the intervention.

### Participants

Recruitment was done online through a banner on a website for adults aged 50 years or older to enable meeting and shared activities and through articles in 8 (regional) newspapers. Older age (being 50 years or older) was the only inclusion criterion for participation. The intervention was not advertised as a loneliness intervention but as an intervention to *benefit more from friendship*. Participation in the intervention was free of charge, and no reward was offered for participation in the study. All communication with the participants was automated, and only if a problem occurred, participants could contact the researcher.

### Measurements

#### Attrition

We assessed the number of lessons participants completed through the management system of the intervention. Completion of the intervention is operationalized as completing the introductory lesson and all 10 substantive lessons. We considered a participant to have dropped out when the number of lessons followed was lower than 10.

#### Loneliness

Loneliness was measured with the De Jong Gierveld Loneliness scale [20]. The 11-item scale consists of a 6-item scale for emotional loneliness and a 5-item scale for social loneliness. The scale includes statements such as “There is always someone I can talk to about my day-to-day problems” for social loneliness and “I miss having a really close friend” for emotional loneliness. Answer categories were “Yes!,” “Yes,” “More or less,” “No,” and “No!” Loevinger coefficient for scale homogeneity was *H*=0.53, and ρ=0.91 for reliability.

#### Ways of Coping

Ways of coping was measured following the method used by Schoenmakers et al [21]. Participants responded to statements related to active and regulative coping with loneliness. Participants were asked whether or not they thought the stated action was suitable for someone who experienced loneliness. Moreover, 3 statements represented active coping (“Attend a course to learn to make and keep friends,” “Go to places or club meetings in order to meet people,” and “Become a volunteer”), and 3 statements represented regulative coping (“Keep in mind that other people are lonely as well, or even more lonely,” “He/she should appreciate the existing contacts with relatives and friends more,” and “Family and friends should point out that he/she must not complain and be realistic”).

#### Intervention Content—Inclusion of Active Elements

The intervention consisted of 2 blocks of 5 lessons of which content differed in activating the participant, but not in topic. The introductory lesson was the same for all participants and introduced some key concepts of the intervention (such as friendship) and let participants reflect on the current state of their network. Subsequently, there was an active and a reflective block. The active block was designed to stimulate participants’ behavior. Participants were given information on the topics and stimulated to actually work on different aspects of friendship mainly through assignments. Participants were invited to renew contact with old friends and initiate small talk with people in the neighborhood. The lessons aimed to educate participants on several aspects regarding social relationships, in order to equip participants with skills to use in different situations. The reflective intervention part consisted of more passive content, which included different stories about friendship. The reflective block stimulated reflection on the 5 topics through existing texts and videos on friendship, for example, a newspaper item on having a holiday by yourself and a comedian talking about cross-sex friendship. Participants were randomized in 2 groups: 1 group started with the active intervention block, followed by the reflective intervention block; the other group followed the blocks of the intervention in a reversed sequence.

#### Engagement—Tempo

The first variable for engagement was the tempo at the start of the intervention, which was the time elapsed between the introductory lesson and the first lesson. Information was obtained through the management system of the intervention.

#### Engagement—Number of Diaries

The second variable used to measure engagement was the number of diaries the participants filled out. Each day, regardless of whether or not participants used the intervention that day, participants received an invitation to fill out a daily diary. The number of diaries participants filled out between the introductory lesson and the first lesson ranged from 0 to 14.

### Other Factors

#### Self-Efficacy

Self-efficacy was measured with a topic-specific measure of self-efficacy. The Social Self-Efficacy Scale refers to the individual’s belief in his or her ability to engage in social contacts [22]. An example of 1 of the 4 items used is as follows: “It is difficult for me to make new friends.” Scores range from 4 to 20; reliability *α*=.70.

#### Information and Communication Technology Proficiency

ICT proficiency was assessed with 2 items, with 1 item asking: “Do you have to ask for help from others when using your computer or mobile phone?” Answer categories were “No,” “Yes, fewer than a couple of times a year,” “Yes, a couple of times a year,” “Yes, a couple of times a month,” “Yes, a couple of times per week,” and “Yes, daily.” A higher score (range 1-6) indicated that more frequent help was needed. In the second item, we asked participants how many types of devices they owned. Categories were desktop, laptop, tablet, smartphone, and smart TV.

### Procedure

We described the differences between 3 categories of participants at baseline: participants who signed up for the intervention and started the lessons, participants who signed up but only completed the baseline questionnaire and the intervention’s introductory lesson but no further lessons, and a third category of participants who signed up, completed the baseline questionnaire, but never started any of the intervention elements. We tested the hypotheses on nonusage attrition by applying survival analysis (Cox regression in IBM SPSS Statistics 25 for Windows) among participants who started the lessons (N=233). The total number of lessons (1-10) was used as time variable. The hazard ratio (HR) and the 95% CI are presented. Tolerance of predictors ranged between .76 and .95.

Hypothesis 1 was tested by adding loneliness at baseline to the survival model. We added variables for the active (hypothesis 2a) and regulative (hypothesis 2b) coping preference to the multivariate model. Confirmatory 2-factor analysis was performed in Mplus [23] for the *ways of coping* measure, using the robust weighted least square estimator [24]. Hypothesis 3 was tested by adding the variable representing the activation by intervention content to the model (active-reflective sequence and reflective-active sequence). Finally, hypothesis 4 was tested by adding the 2 engagement variables to the model: tempo and number of diaries. Due to the relatively small sample size, all hypotheses were tested in bivariate models first, followed by 1 multivariate model. To better understand the meaning of the actual size of the estimated coefficients, we calculated the median survival time in weeks for the 10th and the 90th percentile scores of relevant independent variables. Calculations were made in a multivariate model with the survival procedure in IBM SPSS Statistics 25 for Windows. Continuous variables were categorized according to the percentile scores.

## Results

**Figure 1:**
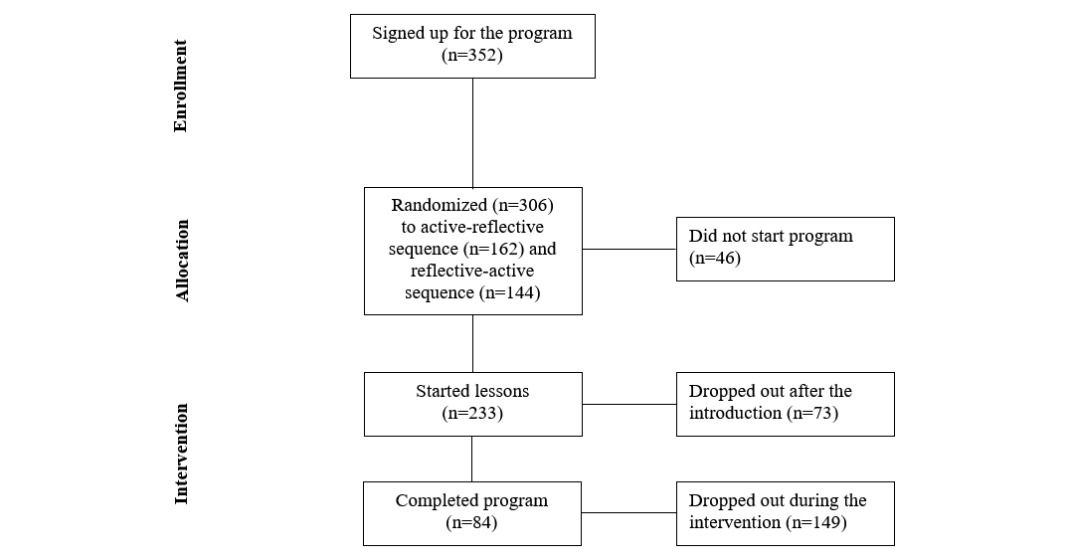
Flowchart of participation in the intervention.

Between April and July 2013, a total of 383 persons signed up for the intervention, of which 6 never provided any data. The baseline questionnaire was filled out by 352 participants, and 313 participants were randomized into 1 of the 2 sequences (Figure 1 provides a flowchart of participation). Most of the participants were female (77.6%; 273/352). Less than half of the participants (42.1%; 148/352) had a partner. The median educational level was 7 on a scale ranging from 1 (primary education) to 9 (university).

**Figure 2:**
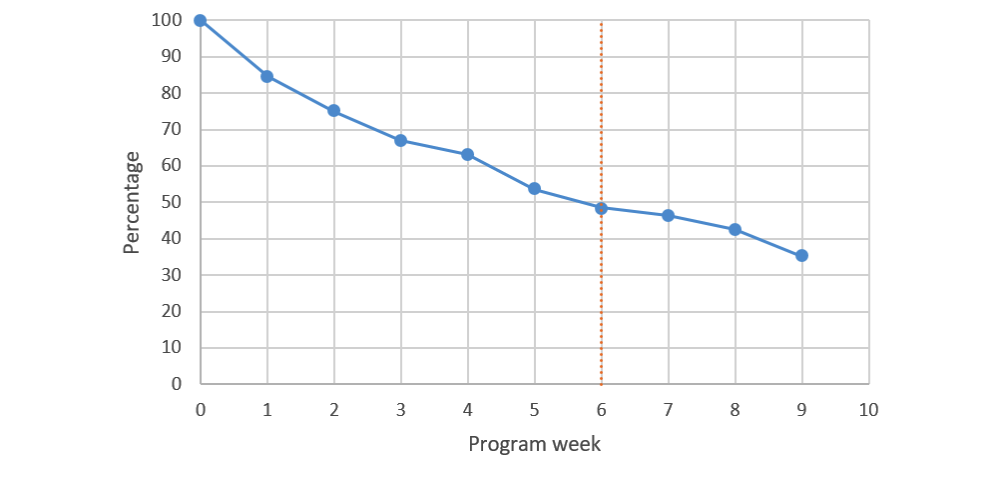
Attrition per program week (n=233). The dotted line represents the median survival time in program weeks.

Of the 352 participants who filled out the baseline questionnaire, 46 participants only provided information at baseline but did not start the intervention, and 306 started the intervention, of which 162 were in the active-reflective and 144 in the reversed sequence. Among the 306 participants who started the intervention, 73 participants did not take part in any of the substantive lessons, but only completed the introductory lesson, leaving 233 participants who followed the substantive lessons. The 233 participants followed on average 6.2 lessons (SD 3.6). Figure 2 shows the percentage of dropouts per program week. The vertical dotted line in Figure 2 indicates the average program weeks the participants completed before dropping out. All 10 lessons were completed by 82 participants (35%; 82/233); 11 of those 82 were study dropouts because they did not fill out the follow-up questionnaire at the end of the intervention. They did, however, remain in the analysis because baseline data and data on time in the intervention were used. The remaining 151 participants (64.8%; 151/233) were nonusage dropouts.

The 2-factor structure of the 6-item questionnaire developed by Schoenmakers et al [21] was confirmed for our data by means of a confirmatory factor analysis (root mean square error of approximation=.00, comparative fit index=1.00, Tucker-Lewis index=1.01; χ^2^_8_=7.7, *P*=.47). The mean social self-efficacy score at baseline was 11.4 (SD 3.0). The mean number of diaries participants filled out between the introductory lesson and the first lesson was 3.67 (SD 2.41). For tempo, a score of 0 indicated that the participant was on track and took 7 days between the 2 lessons. A negative score indicated that the participant took longer than scheduled. The score was calculated by dividing the number of days between the 2 lessons by 7 (indicating 1 week) and was reverse coded (mean −.90 [SD 2.53]; range −19.43 to 0; n=233). Positive scores were not possible because the first lesson became available 7 days after completion of the introductory lesson. With regard to ICT help, a higher score represented more frequent help was needed (mean 2.37 [SD 1.09]). On average, participants owned 2.1 types of devices (SD 1.1).

We compared participants in the 3 phases of nonusage dropout, that is, 46 nonstarters, 73 starters who took the introductory lesson only, and 233 starters with substantive lessons followed (Table 1). The first 2 categories had no follow-up time and thus had no value for tempo. No difference was observed in baseline characteristics for the 3 categories of participation.

**Table 1:** Descriptive statistics for 3 types of participants at baseline.

Variables	Participants who only provided baseline information (n=46)	Participants who only took the introduction (n=73)	Participants who started substantive lessons (n=233)	*F* test (*df*)	Chi-square (*df*)	*P* value
Baseline loneliness (0-11), mean (SD)	7.9 (3.5)	7.5 (3.6)	8.1 (3.0)	1.1 (2, 349)	—^a^	.33
Active coping (0-3), mean (SD)	2.7 (0.6)	2.7 (0.6)	2.7 (0.6)	0.1 (2, 326)	—	.92
Regulative coping (0-3), mean (SD)	1.8 (1.0)	1.6 (0.8)	1.7 (0.9)	0.5 (2, 326)	—	.59
Active-reflective sequence (vs reversed), n (%)	—	33 (45)	129 (55)	—	—	—
Tempo in the first intervention week (−19.4 to 0), mean (SD)	—	—	−0.9 (2.5)	—	—	—
Number of diaries in the first intervention week (0-14), mean (SD)	—	—	3.7 (2.4)	—	—	—
Age (50-88 years), mean (SD)	61.7 (6.0)	63.0 (8.1)	61.7 (7.1)	1.0 (2, 349)	—	.38
Female (vs male), n (%)	31 (67)	59 (81)	183 (79)	—	3.3 (2^b^)	.19
Educational level (1-9), mean (SD)	6.7 (1.8)	6.3 (2.1)	6.5 (2.1)	0.4 (2, 349)	—	.70
Partner (vs no partner), n (%)	20 (43)	37 (51)	91 (39)	—	3.1 (2^b^)	.21
Social self-efficacy (4-20), mean (SD)	11.8 (3.4)	11.8 (2.7)	11.2 (3.0)	1.7 (2, 349)	—	.19
ICT^c^ proficiency: help needed (1-6), mean (SD)	2.4 (1.1)	2.5 (1.0)	2.3 (1.1)	0.9 (2, 349)	—	.40
Number of types of ICT devices (1-5), mean (SD)	1.9 (1.0)	2.2 (1.2)	2.0 (1.1)	1.4 (2, 349)	—	.24

^a^Not applicable.

^b^n=352.

^c^ICT: information and communication technology.

Results from survival analysis among 233 participants who started the lessons are presented in Table 2. In contrast to hypothesis 1, both the bivariate and the multivariate models showed that the baseline level of loneliness did not affect the probability of dropping out.

With respect to hypothesis 2, neither a preference for active nor for regulative coping had an effect on dropout probability in the bivariate analysis. In the multivariate model, however, hypothesis 2a was supported: active coping led to a lower probability of dropping out of the intervention (HR=.73). For participants with high preference for active coping (90th percentile) the median survival time, that is, time that they stay in the intervention, was 8.0 weeks. Participants with low preference for active coping (10^th^ percentile) stayed in the intervention for 5.6 weeks.

Hypothesis 3 on active intervention content was supported in the multivariate model but not in the bivariate model. Participants starting with the active intervention content had a lower probability of dropping out (HR=.71; multivariate model) than other participants. For participants who started with the active intervention content, the median survival time was 7.8 weeks, and participants who started with the reflective content had a median survival time of 5.9 weeks.

To test hypothesis 4 on engagement, we included *tempo* and the number of diaries filled out in the first week of the intervention. The correlation coefficient was .36 (*P*<.001). The hypothesis was supported. Thus, the probability of dropping out was lower when tempo was higher when the participant sticks to the intended pace of the intervention. Participants with high tempo had a median survival time of 9.0 weeks, whereas participants with low tempo had a median survival time of 2.6 weeks. The probability of dropping out was also lower when 1 or more diaries were filled out. Participants who filled out 6 or more diaries (90th percentile) had a median survival time of 9.0 program weeks, and participants who did not fill out diaries (10th percentile) had a median survival time of 2.0 weeks.

Of the other factors, only the number of types of ICT devices affected nonusage attrition in the bivariate analyses. This effect did not show up in the multivariate model. Participants owning more types of ICT devices had a higher probability of dropping out. In the multivariate model, more highly educated participants had a lower probability of dropping out. Participants with a high educational level had a median survival time of 6.1 weeks, and those with a low level had a median survival time of 6.8 weeks.

**Table 2:** Cox regression of nonusage attrition (n=233).

Variables	Bivariate		Multivariate	
	HR^a^ (95% CI)	*P* value	HR (95% CI)	*P* value
Baseline loneliness (0-11)	1.00 (0.95-1.06)	.97	1.00 (0.94-1.07)	.91
Active coping (0-3)	0.81 (0.64-1.03)	.09	0.73 (0.57-0.93)	.01
Regulative coping (0-3)	1.04 (0.84-1.24)	.70	1.01 (0.83-1.22)	.94
Active-reflective sequence (vs reversed)	0.81 (0.59-1.11)	.19	0.71 (0.50-1.00)	.049
Tempo in first intervention week (−19.4 to 0)	0.89 (0.85-0.93)	<.001	0.94 (0.89-1.00)	.049
Number of diaries in first intervention week (0-14)	0.79 (0.73-0.85)	<.001	0.79 (0.72-0.86)	<.001
Age (50-86 years)	0.99 (0.96-1.01)	.26	0.99 (0.97-1.02)	.43
Female (vs male)	0.80 (0.55-1.15)	.23	1.14 (0.75-1.72)	.55
Education (1-9)	0.96 (0.89-1.04)	.32	0.92 (0.84-0.96)	.04
Partner (vs no partner)	1.19 (0.86-1.65)	.29	0.95 (0.66-1.36)	.77
Social self-efficacy (4-20)	1.01 (0.96-1.06)	.77	0.99 (0.93-1.06)	.84
ICT^b^ proficiency: help needed (1-6)	0.93 (0.80-1.07)	.29	0.96 (0.82-1.12)	.59
Number of types of ICT devices (1-5)	1.16 (1.00-1.34)	.048	1.14 (0.97-1.35)	.12

^a^HR: hazard ratio.

^b^ICT: information and communication technology.

## Discussion

This study aimed to gain insight into the factors affecting attrition in an online loneliness intervention. The participants in the oFEP suffered from loneliness varying in intensity so that they form an appropriate sample to study the extent to which severity of the problem affects attrition. There was no support for hypothesis 1 that participants with more severe loneliness remain in the intervention longer than mildly lonely participants. Coping style affected attrition. People with a preference for active coping, who thus are more motivated to tackle the loneliness problem, stayed in the intervention longer (hypothesis 2a; and hypothesis 2b did not find support). Receiving content focused on active coping first (as opposed to reflective content; hypothesis 3) also increased adherence. The effect of engagement with the intervention (hypothesis 4) turned out to be the most important of the factors studied. Participants who were more engaged with the intervention, meaning they participated in the lessons at the intended pace and filled out diaries, were less likely to drop out of the intervention. Finally, we also explored the association between several other, mainly personal, characteristics and attrition. More educated participants tended to stay in the intervention longer. This could be understood from the format of the lessons. Higher education may enable participants to read and comprehend written text better, and hence these participants adhere more to the intervention.

These findings imply that, when trying to increase adherence to an online intervention, it is not necessary to select participants based on the severity of their problem. It seems to be beneficial to pay attention to coping preference and stimulate more active coping. For future interventions, it may be useful to try to persuade people to engage in more active coping, even when this is not their preferred coping style. This approach complies with the notion that it takes a lot of effort to tackle problems such as loneliness and with the finding that loneliness interventions are often not successful [25]. Moreover, the success of efforts to combat loneliness is not always immediately apparent [26]. Future interventions may attempt to stimulate participants even more to engage in active coping, for example, with testimonials that focus on the benefits of engaging in active coping, or by pointing out that the extra effort that active coping requires may pay off. Lucas et al [27] suggested that it is possible to break through regulative coping preferences and passive social behavior. Priming lonely individuals to engage in more positive behavior can reduce their focus on cautious social behavior. Our finding that engagement affects attrition provides especially valuable insight for future interventions. It allows intervention developers to intervene with additional resources as soon as participants seem to lower their engagement. For example, in the oFEP, we can send an extra message as a reminder to participants who do not participate in the second lesson within 10 days. Furthermore, the importance of following the lessons of the intervention at the intended pace of 1 per week can be stressed throughout the intervention. A word of caution here is that there might be between-person differences in which principles work best to increase engagement [28]. What works for or is preferred by 1 participant, might not be preferred by another. It seems that some level of personalization of the intervention is required, but further research on this topic is needed. Instead of increasing engagement of participants, an intervention developer can also use participants’ engagement as a selection criterion, for example, to direct the limited resources to only those participants who are most likely to complete the intervention. Selection can, in that case, be done by means of a brief preintervention.

There are 2 design issues in this study that need discussion. The questionnaires were included in the intervention and not presented as separate study. Completion of certain parts of the program triggered the release of the questionnaires, and only after completion of the questionnaire, the participants could continue with the intervention. By making these design choices, we lost the possibility to distinguish nonusage attrition from dropout attrition. Including the questionnaires in the intervention may have increased participants burden and could thus potentially affect the results. A limitation is that we were not able to assess how the loneliness of participants who dropped out from the intervention developed over time. It can be that they had already benefited from the lessons and were able to reduce their loneliness, similar to participants who completed the intervention. If this is the case, the intervention was successful and participant’s nonusage attrition is a conceivable choice. However, continued participation might contribute to a further strengthening of the person and his situation. Furthermore, this study only looked at baseline characteristics as factors influencing attrition. Unfortunately, not all factors of interest were observed at least weekly, preventing the inclusion of time-varying characteristics into the analysis. The severity of the problem and the engagement with the intervention may change in the course of the intervention. With respect to the measurement of the variable *tempo*, we limited it to the first week and did not extend it to the whole intervention. The latter is problematic for participants who dropped out of the intervention before completion of the intervention. Furthermore, we reasoned that tempo in the first week of the intervention indicated the initial commitment of the participant to the intervention. Finally, by conducting and reviewing only 1 intervention, we did not test the importance of design characteristics. The review of Murray et al [9] shows that a sound theoretical foundation [29], tailoring [16], and the use of prompts [30] result in an intervention design with improved participants’ adherence to the intervention.

In conclusion, we observed that active coping prevents attrition. Eysenbach [6] argues that high attrition is a weakness of all self-guided online interventions. However, our study suggests that improvement is possible. Future online loneliness interventions might try to lower attrition by stimulating active behavior, for example, by offering a variety of exercises and an active approach toward participants with a slow pace in conducting intervention activities.

## Abbreviations

HR: hazard ratio
ICT: information and communication technology
oFEP: online Friendship Enrichment Program
